# Immune checkpoint inhibitors plus trastuzumab and chemotherapy for the treatment of advanced HER2-positive gastric and gastroesophageal junction cancers: a systematic review and meta-analysis

**DOI:** 10.3389/fimmu.2026.1832353

**Published:** 2026-07-01

**Authors:** Hongjie Zhan, Hongbo Zhang, Caijuan Tian, Pengfei Liu, Weilin Sun

**Affiliations:** 1 Department of Gastric Surgery, Tianjin Medical University Cancer Institute & Hospital, National Clinical Research Center for Cancer, Tianjin’s Clinical Research Center for Cancer, Tianjin Key Laboratory of Digestive Cancer, Tianjin, China; 2 Tianjin Marvel Medical Laboratory, Tianjin Marvelbio Technology Co., Ltd, Tianjin, China; 3 Institute of Basic Research, Tianjin Academy of Traditional Chinese Medicine, Tianjin Academy of Traditional Chinese Medicine Affiliated Hospital, Tianjin, China

**Keywords:** gastric cancer, HER2, immune checkpoint inhibitors, trastuzumab, treatment strategies

## Abstract

**Background:**

HER2-positive gastric and gastroesophageal junction (GEJ) cancers have poor prognosis despite standard trastuzumab-based chemotherapy. Immune checkpoint inhibitors (ICIs) may enhance therapeutic efficacy when combined with trastuzumab.

**Methods:**

A systematic review and meta-analysis were conducted following PRISMA guidelines. PubMed, Cochrane Library, Embase, and Scopus were searched up to December 29, 2025. Studies evaluating ICIs plus trastuzumab and chemotherapy versus trastuzumab-based therapy alone were included. Kaplan–Meier curves were digitized using WebPlotDigitizer and reconstructed to individual participant data (IPD). Pooled survival outcomes, objective response rate (ORR), disease control rate (DCR), and safety were analyzed.

**Results:**

Six studies including 1,097 patients were included. ICIs plus trastuzumab and chemotherapy significantly improved median progression-free survival (10.4 vs. 8.27 months; HR 0.6951, p<0.0001) and overall survival (20.4 vs. 17.2 months; HR 0.8104, p=0.004) compared with control. ORR (OR 1.85, p<0.00001) and DCR (OR 2.46, p=0.006) were also superior. Grade ≥3 adverse events were similar between groups.

**Conclusions:**

ICIs combined with trastuzumab and chemotherapy improve survival and response rates in HER2-positive gastric/GEJ cancers with manageable toxicity, supporting their integration into treatment strategies.

**Systematic review registration:**

https://www.crd.york.ac.uk/prospero/, identifier CRD420261280958.

## 1 Introduction

Based on incidence and mortality data from GLOBOCAN 2022, it is estimated that a total of 15.6 million gastric cancer cases will occur worldwide among populations born between 2008 and 2017 across 185 countries (1). Approximately 75% of these cases are expected to be concentrated in Asia, and in certain regions, the future burden of gastric cancer may be up to six times higher than the estimates reported for 2022 (1, 2). About 60% of gastric cancer cases are diagnosed at the regional or distant stage, for which the 5-year relative survival rates are only 31% and 6%, respectively (3). Over 40% of patients with esophagogastric junction tumors experience recurrence within the first year after potentially curative surgery, and post-recurrence survival is typically less than 6 months due to the limited effectiveness of available treatments, including palliative chemotherapy, radiotherapy, or surgical resection of isolated lesions (4). In HER2-positive gastric and gastroesophageal junction (GEJ) cancers, combining trastuzumab with fluoropyrimidine- and capecitabine-based chemotherapy has shown significant antitumor activity and is now regarded as the standard of care (5, 6). For patients with advanced gastric or gastroesophageal junction (GEJ) cancer whose tumors are either IHC 2+ and FISH positive or IHC 3+, trastuzumab should be considered, as it has demonstrated a survival benefit and is well tolerated (7). This regimen achieves an objective response rate (ORR) of 44.3% (95% CI: 37.8–50.9), a median progression-free survival (PFS) of 5.6–7.1 months, and a median overall survival (OS) of 14.0–20 months (6).

Immune checkpoint inhibitors (ICIs) targeting PD-1 and PD-L1 have transformed the treatment of advanced and metastatic gastric and gastroesophageal junction (GC/GEJ) cancers, with large phase III trials—including CheckMate 649, KEYNOTE-859, and GEMSTONE-303—establishing PD-1/PD-L1 blockade combined with chemotherapy as a new standard that significantly improves overall survival (OS) (8–11). While, tislezumab combined with trastuzumab and standard chemotherapy for HER2-positive GC/EGJ have good clinical benefit (12). The addition of pembrolizumab to trastuzumab and chemotherapy significantly prolonged progression-free survival (PFS) and improved objective response rate (ORR), with durable responses, particularly in patients with a programmed death-ligand 1 (PD-L1) combined positive score (CPS) of ≥1 (13). Although immunotherapies, particularly ICIs, improve survival in gastric cancer, patients with diffuse-type GC exhibit poor responsiveness due to the cold tumor immune microenvironment shaped by their histological and molecular characteristics (14). HER2-targeted antibody–drug conjugates have emerged as an important therapeutic strategy in gastric and gastroesophageal junction cancers, with trastuzumab deruxtecan demonstrating superior efficacy over conventional chemotherapy after trastuzumab failure, while ongoing development of next-generation ADCs continues to expand treatment options for HER2-positive disease (15). The KEYNOTE-811 study demonstrated that in HER2-positive gastric or gastroesophageal junction cancer, pembrolizumab combined with trastuzumab and chemotherapy achieved an objective response rate of up to 74.4%, suggesting that PD-1 inhibition may improve clinical outcomes by enhancing therapeutic efficacy and delaying the development of resistance (16). Targeted therapy, particularly trastuzumab, has significantly improved survival in HER2 positive gastric cancer, but its long term efficacy is limited by primary and acquired resistance driven by tumor heterogeneity and activation of downstream HER2 signaling pathways (17). Although these findings highlight the therapeutic potential of combining trastuzumab with immune checkpoint inhibitors, the incorporation of this strategy into standard treatment paradigms for HER2-positive tumors remains under active investigation.

New anti-HER2 agents, such as trastuzumab deruxtecan (T-DXd; DS-8201a) and disitamab vedotin (RC48), have demonstrated substantial therapeutic advances in gastric and gastroesophageal junction cancers, underscoring a rapidly evolving treatment landscape and the potential for integration with immune checkpoint inhibitors (ICIs) (18). However, a comprehensive synthesis of current evidence, including interim randomized data and real world studies, evaluating the efficacy and safety of combining immune checkpoint inhibitors with trastuzumab in HER2 positive gastric and gastroesophageal junction cancer remains lacking. With recent regulatory approvals and multiple ongoing international trials, an updated and consolidated evaluation is urgently needed to inform clinical practice. Therefore, this study aims to assess the efficacy and safety of combined immune checkpoint inhibitor and trastuzumab therapy in patients with HER2 positive gastric cancer.

## 2 Methods

This systematic review and meta-analysis was performed in according with Preferred Reporting Item for Systematic Reviews and Meta-Analysis guidelines. The research protocol was registered with the International Prospective Register of Systematic Reviews (PROSPERO 2026 CRD420261280958).

### 2.1 Search strategy

PubMed, Cochrane Library, Embase, and Scopus were systematically searched on December 29, 2025. Search terms included “gastric cancer”, “stomach cancer”, “gastroesophageal junction cancer”, “gastric carcinoma”, “HER2”, “HER2-positive”, “trastuzumab”, “programmed cell death protein 1”, “PD-1 inhibitor”, and “PD-L1 inhibitor”, which were combined using the Boolean operators “AND” and “OR”. The search was conducted across PubMed/MEDLINE, Embase, and Cochrane CENTRAL, ensuring broader coverage of peer-reviewed literature. After duplicate records were removed, two authors (Hongjie Zhan and Hongbo Zhang) independently screened the titles and abstracts. Full texts of potentially eligible studies were then assessed to determine final inclusion, with any discrepancies resolved through discussion. A third author (Pengfei Liu) reviewed titles when necessary to adjudicate unresolved disagreements. In addition, the reference lists of relevant publications were manually examined to identify any additional eligible studies.

Inclusion criteria:

1. Patients diagnosed with gastric cancer;
2. HER2 status confirmed according to pathological testing results
3. Studies comparing immune checkpoint inhibitor–based therapy (e.g., PD-1/PD-L1 inhibitors) combined with trastuzumab and chemotherapy versus trastuzumab plus chemotherapy alone;
4. Studies reporting at least one of the following outcomes: progression-free survival (PFS), overall survival (OS), and/or safety data;
5. Randomized clinical trials, retrospective studies, observational studies, as well as real-world and clinical studies.

Exclusion criteria:

1. Studies lacking an appropriate control or comparator group;
2. Conference abstracts, reviews, case reports, book chapters, comments, and short surveys;
3. Studies not published in English;
4. Studies using treatment strategies that did not include trastuzumab.

### 2.2 Literature selection process

The literature search was conducted independently by two reviewers (Hongjie Zhan and Caijuan Tian) using a predefined search strategy. After duplicate records were removed, titles and abstracts were screened, and studies deemed potentially eligible underwent full-text assessment. All retrieved articles were evaluated against the predefined inclusion criteria. Any disagreements between the reviewers were resolved through consultation with a third author (Weilin Sun).

### 2.3 Data extraction and quality assessment

The following data was extracted from all eligible articles: (1) first author name; (2) year of publication; (3) number of participants; (4) overall survival (OS), defined as the time from treatment initiation to death from any cause; (5) progression-free survival (PFS), defined as the time from treatment initiation to disease progression or death from any cause; (6) objective response rate (ORR), defined as the proportion of patients achieving a complete or partial response; (7) disease control rate (DCR), defined as the proportion of patients achieving complete response, partial response, or stable disease; and (8) safety outcomes, including adverse events of all grades and grade ≥3, assessed according to the CTCAE V6 (19). The methodological quality of the included studies was evaluated using the RoB 2 tool for randomized controlled trials and the ROBINS-E tool for non-randomized studies. Risk-of-bias assessments were conducted independently by two reviewers (Hongbo Zhang and Caijuan Tian), and any disagreements were resolved through discussion with a third reviewer (Hongjie Zhan). This procedure minimized potential assessment bias and ensured the objectivity and reliability of the evaluation (20).

### 2.4 Statistical analysis

Statistical analyses were performed using the “meta” and “IPDfromKM” packages in R statistical software (version 4.5.1). Statistical heterogeneity across studies was assessed using the I² statistic, with values of 30–60% indicating moderate heterogeneity, 50–90% substantial heterogeneity, and 75–100% considerable heterogeneity. All statistical analyses were conducted using Review Manager (RevMan) version 5.4 (Nordic Cochrane Centre, Copenhagen, Denmark) and SPSS version 27.

For time-to-event outcomes, including OS and PFS, raw coordinate data (time and survival probability) were extracted from published Kaplan–Meier curves of individual studies using WebPlotDigitizer. These extracted data were then integrated with the reported numbers at risk at corresponding time points to reconstruct individual participant data, which were pooled across studies to generate a combined study dataset. A pooled Kaplan–Meier curve was subsequently generated, and median survival, hazard ratios, and corresponding 95% confidence intervals were estimated using GraphPad. For categorical variables, the chi-square test was used to assess significance, and for all outcomes, statistical significance was set at a two-sided P value < 0.05.

## 3 Results

**Figure 1:**
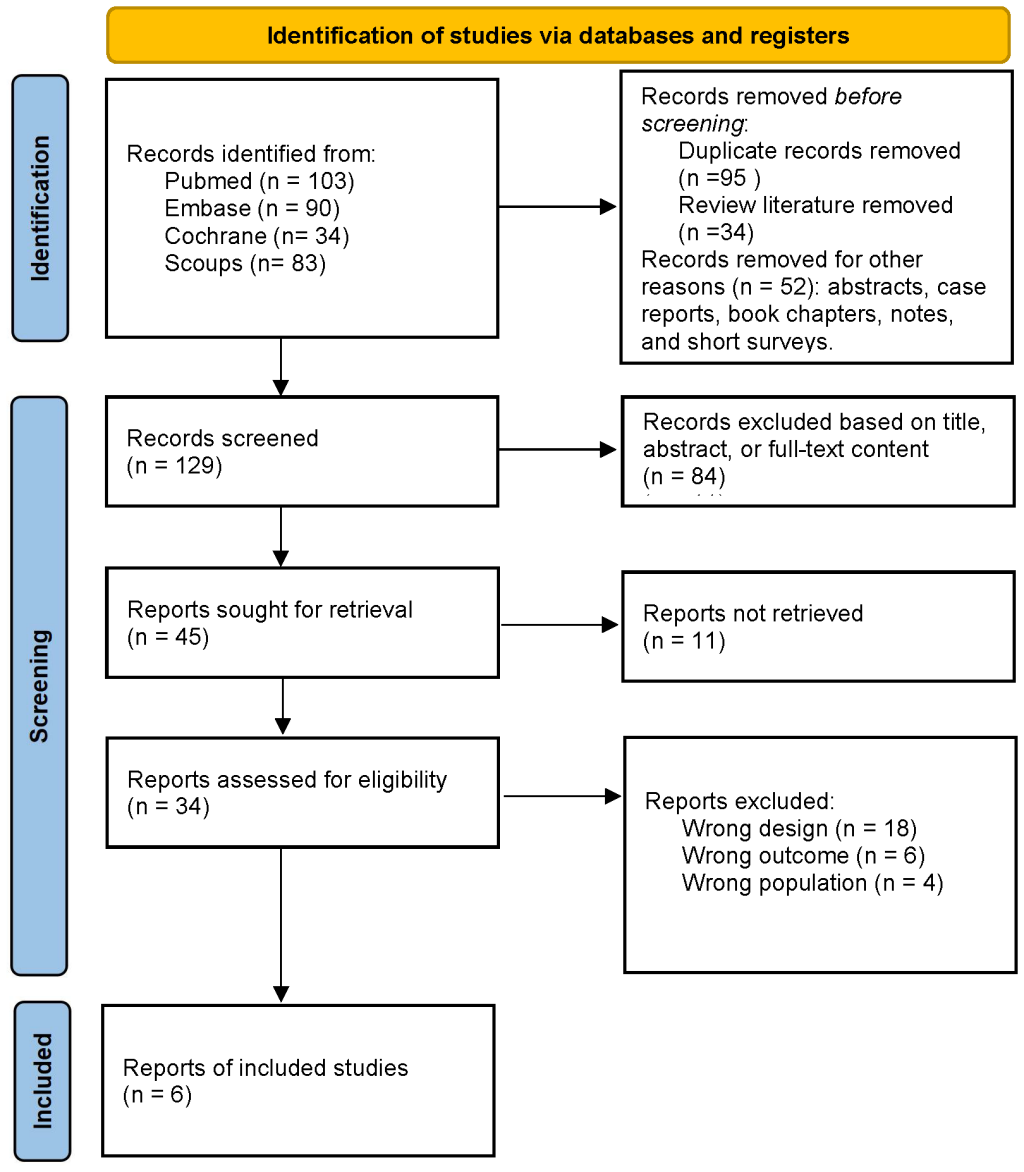
Preferred reporting items for systematic reviews and meta-analyses (PRISMA) flow diagram with systematic review of detailed searches.

A total of 310 potentially relevant articles were identified through searches in PubMed, EMBASE, Cochrane, and Scopus in December 2025. After removing 95 duplicate records, 34 review articles, and 52 publications excluded based on type (e.g., abstracts, case reports, comments), 129 articles remained. Subsequently, based on title and abstract screening and full-text—considering whether the study design involved immune checkpoint therapy combined with trastuzumab and chemotherapy for gastric cancer and whether a control group was included—6 high-quality studies meeting the inclusion criteria were ultimately selected (**Figure 1**; **Table 1**). A total of 1,097 patients from these 6 studies were included in the analysis (**Table 1**). The risk-of-bias assessments for randomized controlled trials and non-randomized studies are presented in **Supplementary Table 1**. Pooling PD-1 and PD-L1 inhibitors without appropriate stratification could introduce clinical heterogeneity. However, after carefully reviewing all eligible studies, we confirm that the six treatment regimens included in our analysis—such as pembrolizumab, camrelizumab, and sintilimab —are all anti–PD-1 monoclonal antibodies. No PD-L1 inhibitors were included in the final pooled analysis. Therefore, the intervention class is mechanistically homogeneous, as all agents target the PD-1 receptor and share a comparable mode of immune checkpoint blockade.

**Table 1:** Clinical and demographic characteristics of studies included in the meta-analysis.

Author	Year	Study design	Author states	Sample size	Age	Sex (male/female)	Intervention/control	Intervention	Control	Follow-up (months)	Median PFS (intervention group/control group)	Median OS (intervention group/control group)
Zhang S, et al. (11)	2025	Retrospective	China	104	64	81/23	54/50	Pembrolizumab/Camrelizumab/Sintilimab+trastuzumab+chemotherapy	trastuzumab+chemotherapy	14.6	9.3/6.9	–
Xu M, et al.	2022	Retrospective/ChiECRCT20220008	China	41	–	36/5	28/13	Camerelizumab+trastuzumab+chemotherapy	trastuzumab+chemotherapy	10	10.1/6.0	18.4/13.2
Liu Z, et al.	2025	Retrospective	China	103	–	77/36	46/57	Sintilimab+trastuzumab+chemotherapy	trastuzumab+chemotherapy	14.3	9.4/7.4	16.4/14.2
Deng T, et al.	2023	Retrospective	China	56	62/55.5	40/16	30/26	anti-PD-1 antibody +trastuzumab	trastuzumab+chemotherapy	17.05/23.65	16.2/14.5	28.1/31.6
Janjigian YY, et al. (26)	2023	RCT/NCT03615326	USA	698	62/63	564/134	350/348	Pembrolizumab + trastuzumab + chemotherapy	trastuzumab+chemotherapy	54	10/8.1	20/16.9
Zhang L, et al. (29)	2025	Retrospective	China	95	–	69/26	32/63	PD-1 inhibitor+trastuzumab+chemotherapy	trastuzumab+chemotherapy	26	15.57/7.57	24.67/16

Of the 1,097 patients, 540 received the combination of trastuzumab, chemotherapy, and immune checkpoint inhibitors. In the intervention group, median progression-free survival (PFS) ranged from 9.3 to 15.57 months and median overall survival (OS) ranged from 16.4 to 28.1 months, whereas in the control group, median PFS ranged from 6.0 to 14.5 months and median OS from 13.2 to 31.6 months. Additional characteristics from the included studies, including study design, median age, sex distribution, intervention and control details, and follow-up duration, are summarized in **Table 1**.

**Figure 2:**
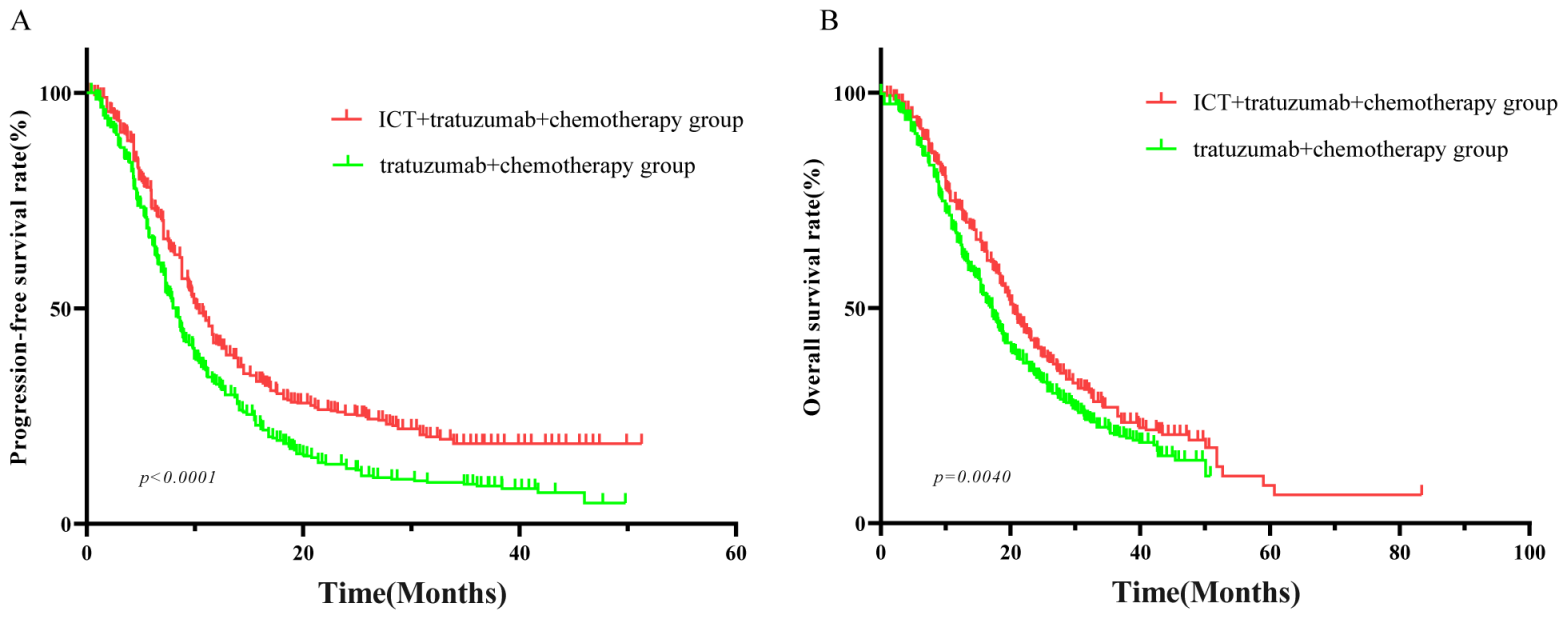
Kaplan-Meier curves for immune checkpoint therapy combined with trastuzumab and chemotherapy vs. trastuzumab and chemotherapy alone. **(A)** Progression-free survival in patients with HER2-positive gastric or gastro-oesophageal adenocarcinoma; **(B)** Overall survival in patients with HER2-positive gastric or gastro-oesophageal adenocarcinoma.

The method of reconstructing Kaplan−Meier curves from published plots has been widely used and validated (21, 22), which enables approximation of individual time−to−event data when true patient−level data are unavailable. After reconstructing the Kaplan–Meier curves from the six included studies and restoring the underlying coordinate data, pooled survival curves for the two groups were generated using GraphPad. In the intervention group, the median progression-free survival (PFS) was 10.40 months compared with 8.27 months in the control group (HR 0.6951; 95% CI: 0.6012–0.8037; p < 0.0001). Median overall survival (OS) in the intervention group was 20.4 months versus 17.2 months in the control group (HR 0.8104; 95% CI: 0.7010–0.9369; p = 0.0040) (**Figure 2**).

**Figure 3:**
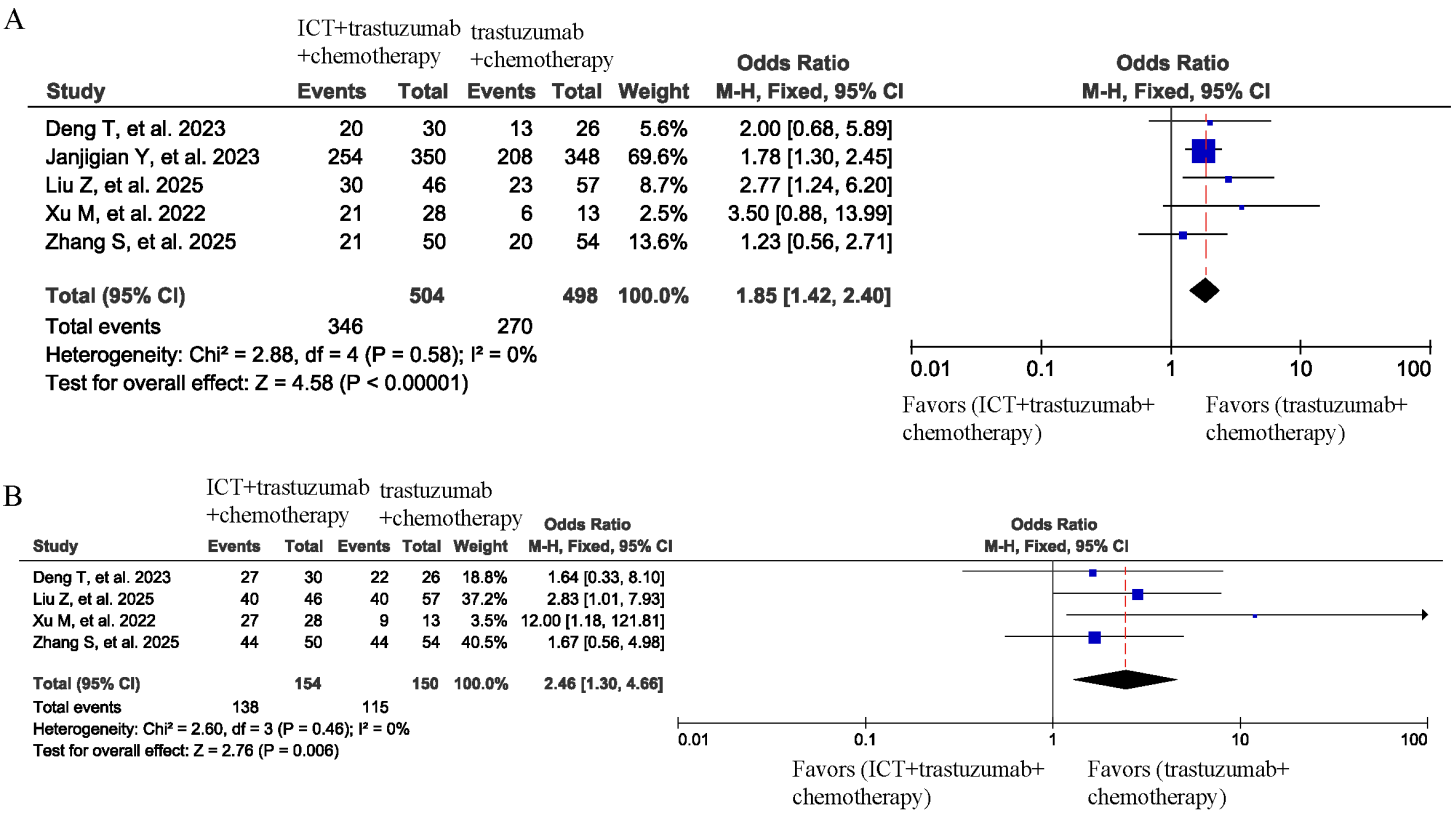
Forest plots of odds ratios (HR) with 95% confidence intervals (CI) for **(A)** Objective Response Rate and **(B)** Disease Control Rate in patients with HER2-positive gastric or gastro-oesophageal adenocarcinoma.

We then compared the efficacy of the two groups using objective response rate (ORR) and disease control rate (DCR). As shown in **Figure 3**, the combination of immune checkpoint therapy, trastuzumab, and chemotherapy demonstrated superior treatment outcomes. Among patients with gastric cancer receiving this combination, ORR was significantly higher in the intervention group (OR 1.85; p < 0.00001; **Figure 3A**). Similarly, DCR was also significantly improved in the intervention group compared with the control group (OR 2.46; p = 0.006; **Figure 3B**).

The incidence of diarrhea, hypothyroidism, abnormal liver function, and pneumonia was significantly higher in the intervention group than in the control group (**Table 2**). However, no significant differences were observed between the two groups for adverse events of grade ≥ 3. Therefore, the overall risk in the intervention group was manageable, indicating that the combination of immune checkpoint therapy, trastuzumab, and chemotherapy can be safely used in the treatment of HER2-positive gastric cancer.

**Table 2:** Incidence of adverse events.

Adverse events	ICT+trastuzumab+chemotherapy group	trastuzumab+chemotherapy group	Risk rate (95%CI)	P value
Non-hematologic system				
Decreased appetite	101/426	99/426	1.02 (0.80–1.30)	0.872
Diarrhea	191/456	163/479	1.23 (1.04–1.45)	0.013
Fatigue	74/396	61/403	1.23 (0.91–1.68)	0.181
Hypokalemia	11/76	6/83	2.00 (0.78–5.15)	0.14
Hypothyroidism	69/536	28/559	2.57 (1.68–3.92)	<0.001
Nausea and vomiting	280/486	268/505	1.09 (0.97–1.21)	0.15
Neutropenia	115/412	103/412	1.12 (0.89–1.40)	0.343
Rash	18/454	8/442	2.19 (0.96–4.99)	0.055
Stomatitis	49/442	42/416	1.10 (0.74–1.62)	0.638
Abnormal liver function	25/106	15/133	2.09 (1.16–3.76)	0.011
Pneumonia	27/476	10/483	2.74 (1.34–5.60)	0.004
Hand-foot syndrome	16/106	11/133	1.83 (0.88–3.76)	0.098
Hematologic system				
Anemia	128/412	132/435	1.02 (0.84–1.25)	0.82
Thrombocytopenia	142/462	155/489	0.97 (0.80–1.17)	0.749
Leukopenia	24/62	23/89	1.50 (0.94–2.40)	0.093
Neurotoxicity	169/486	152/505	1.16 (0.96–1.38)	0.116
Bone marrow suppression	40/74	29/70	1.30 (0.92–1.85)	0.13
Adverse events Grade≥3	322/536	295/536	1.09 (0.98–1.21)	0.095

## 4 Discussion

In this systematic review and meta-analysis, six studies comprising a total of 1,097 patients with advanced HER2-positive gastric or gastroesophageal junction adenocarcinoma were included. Compared with trastuzumab-based therapy alone, the addition of immune checkpoint inhibitors to trastuzumab significantly prolonged progression-free survival (10.4 months vs. 8.27 months; p < 0.0001) and overall survival (20.4 months vs. 17.2 months; p = 0.004), and was also associated with significant improvements in objective response rate (p < 0.00001) and disease control rate (p = 0.006). Trastuzumab may enhance immunotherapy by inducing antibody-dependent cellular cytotoxicity, promoting tumor antigen release and presentation, activating tumor-specific T-cell responses, and synergizing with immune checkpoint inhibitors through interferon-γ–mediated upregulation of PD-L1 in the tumor microenvironment (23).

Programmed cell death-1 (PD-1)/programmed death-ligand 1 (PD-L1) checkpoint inhibitors are widely used in the treatment of many solid tumors, as the interaction between PD-L1 and PD-1 expressed on the surface of T cells suppresses antitumor T-cell activity, enabling tumor cells to evade immune surveillance (24). The inhibitory PD-1 pathway plays a central role in the differentiation of tumor-reactive stem-like T cells by fine-tuning TCR signal input, thereby enabling the selective expansion of high-affinity TCR stem-like clones as a renewable source of effector cells; in contrast, PD-1 blockade disrupts this regulatory tuning, leading to terminal differentiation or apoptosis of the most avid antitumor stem-like T cells (25).

In the KEYNOTE-811 trial (26), Asian patients appeared to derive less OS benefit from pembrolizumab treatment (HR 1.05, 95%CI: 0.77–1.43; n = 240), compared with non-Asian patients who demonstrated a clear survival benefit (HR 0.72, 95%CI: 0.59–0.87; n = 456). A similar pattern was observed for PFS, where the Asian subgroup showed a more modest improvement (HR 0.85, 95%CI: 0.62–1.16), whereas non-Asian patients experienced a more pronounced benefit (HR 0.69, 95%CI: 0.56–0.84). These findings suggest potential heterogeneity in treatment effect across populations. Several hypotheses may explain this observation, including differences in tumor immune microenvironment, variations in subsequent lines of therapy after disease progression, differences in baseline clinical characteristics, and limited statistical power of subgroup analyses within randomized trials. Our meta-analysis, which predominantly includes Asian real-world studies, provides complementary evidence suggesting that the addition of immune checkpoint inhibitors to trastuzumab-based chemotherapy may still be associated with clinically meaningful survival benefits in Asian patients. This may indicate that the neutral OS signal observed in the KEYNOTE-811 Asian subgroup could reflect methodological limitations or population-specific trial factors rather than a true absence of biological benefit.

HER2 amplification and PD-L1 CPS appear to provide independent but complementary molecular information in advanced gastric cancer, with PD-L1 CPS reflecting immune activity and genomic instability, supporting their combined use to guide personalized targeted and immunotherapy strategies (27). The KEYNOTE-012, KEYNOTE-059, KEYNOTE-062, and CheckMate-649 studies demonstrated that a positive PD-L1 combined positive score (CPS) is an important clinical predictor of improved efficacy for patients receiving combined anti–PD-1 therapy and cytotoxic chemotherapy (28). Furthermore, although PD-L1 CPS has emerged as an important predictive biomarker in KEYNOTE-811 (26), standardized CPS assessment is not universally available in routine clinical practice. Consequently, our pooled analysis may reflect treatment outcomes in a more pragmatic “CPS-agnostic” setting and therefore provide information that is directly relevant to real-world clinical decision-making. Amplification or overexpression of human epidermal growth factor receptor 2 (HER2) occurs in approximately 20% of advanced gastric or gastroesophageal junction adenocarcinomas. While the addition of the anti–programmed cell death-1 (PD-1) antibody pembrolizumab to treatment in HER2-negative advanced gastric cancer did not significantly improve efficacy, dual blockade of PD-1 and HER2 in PD-1–positive patients achieved an objective response rate (ORR) of 74.4%, representing a 22-percentage-point increase compared with trastuzumab plus chemotherapy alone (16). PD-L1 serves as a key predictive biomarker for improved survival in gastric cancer patients receiving trastuzumab-based therapy, and combined treatment with PD-1 inhibitors, trastuzumab, and chemotherapy significantly prolongs progression-free survival (PFS) and overall survival (OS) in HER2-positive patients (29). Additionally, the phase II clinical trial ChiCTR2200058732 demonstrated that sintilimab combined with trastuzumab and chemotherapy is effective in treating HER2-positive gastric cancer, with 50% of patients achieving a complete response (CR) and 55% achieving a partial response (PR) (30). The KEYNOTE-811 study demonstrated that PD-1 blockade combined with HER2-targeted therapy and chemotherapy in metastatic HER2-positive gastric cancer achieved an objective response rate (ORR) of 77.5%, a disease control rate (DCR) of 100%, and a complete response (CR) rate of 15.0%. The regimen was well tolerated, with no treatment-related grade ≥4 adverse events or deaths reported (31). Although this underscores the need for further research in this area, our meta-analysis supports the safety of this combination regimen, as no significant increase in the incidence of severe adverse events (grade ≥3) was observed, except for hypothyroidism, diarrhea, abnormal liver function, and pneumonia, which is consistent with most published reports (30, 31). Moreover, hematologic and gastrointestinal toxicities were not significantly elevated, indicating that this combination therapy is a promising treatment option and could be considered for integration into therapeutic strategies for advanced gastric cancer.

This study included a limited number of articles, with some being single-arm or heterogeneous in design, which may affect the generalizability of the findings. Differences in patient populations, treatment regimens, PD-L1 assessment, and follow-up across studies may also introduce heterogeneity. Additionally, due to the limited number of available studies and the heterogeneity of treatment regimens, we were unable to perform a subgroup analysis based on individual PD-1 inhibitors, and whether different PD-1 inhibitors confer distinct therapeutic benefits remains unclear. Reconstructed Kaplan–Meier individual data may slightly deviate from the original data, and long-term survival outcomes and late-onset adverse events were not consistently reported. These factors suggest that, while the results support the efficacy and safety of combining immune checkpoint inhibitors with trastuzumab and chemotherapy, further large-scale, high-quality randomized trials are needed for confirmation.

## Data Availability

The original contributions presented in the study are included in the article/**Supplementary Material**. Further inquiries can be directed to the corresponding authors.
